# A128 PATIENT CHARACTERIZATION AND ASSESSMENT OF REMISSION RATES IN CHILDREN WITH EOSINOPHILIC ESOPHAGITIS AFTER INITIAL TREATMENT AND 1-YEAR POST-DIAGNOSIS

**DOI:** 10.1093/jcag/gwab049.127

**Published:** 2022-02-21

**Authors:** S Da Silva, K Bortolin, N Afzal, P Marcon, J Hulst

**Affiliations:** 1 ^1.^ University of Toronto Temerty Faculty of Medicine, Toronto, ON, Canada; 2 ^2.^ The Hospital for Sick Children, Division of Gastroenterology, Hepatology and Nutrition, Toronto, ON, Canada, Toronto, ON, Canada; 3 ^3.^ The Hospital for Sick Children, Division of Gastroenterology, Hepatology and Nutrition, Toronto, ON, Canada, Toronto, ON, Canada

## Abstract

**Background:**

Eosinophilic esophagitis (EoE) is a chronic immune- or antigen-mediated esophageal disorder characterized by symptoms of esophageal dysfunction and eosinophil-predominant inflammation. With incidence rates rapidly increasing, EoE is the leading cause of food bolus impaction and the second leading cause of chronic esophagitis. The treatment of EoE includes both induction and maintenance therapy to prevent complications including esophageal fibrosis and strictures. Despite significant advances in the diagnosis and treatment of EoE, the natural history and long-term management of the disease remains poorly understood.

**Aims:**

To describe the clinical characterization of children with EoE, and to assess remission rates after initial treatment and 1-year post-diagnosis.

**Methods:**

In this ongoing, single-center retrospective study, the electronic medical charts of children newly diagnosed with EoE between January 2017-March 2021 participating in the EoE-AHEAD Registry study were reviewed. Children aged 6 months-18 years with a confirmed diagnosis of EoE based on consensus guidelines and receiving outpatient care at the SickKids EoE clinic were included.

**Results:**

A total of 37 children with a median age of 11 years (IQR: 4.5, 12.5) were included, the majority of which were male (86.5%). Demographic and clinical characteristics are shown in **Table 1**. On initial treatment, 2.7% of children were on an elemental diet, 18.9% on a food elimination diet, 40.5% on proton-pump inhibitor (PPI) therapy, 16.2% on topical steroids, 8.1% on systemic steroids, and 13.5% on a combination therapy. Of the 30 children with a second endoscopy (mean interval since diagnosis of 6.1 months (3.9)), 30% achieved histological remission after the initial treatment (see **Figure 1**). Of the 14 children with a one-year follow-up endoscopy, 14.2% were in remission, 3 of which had relapsed. No significant differences in treatment modality were found between those who achieved remission and those who did not.

**Conclusions:**

This is the first Canadian registry study to examine the longitudinal outcomes of EoE in a pediatric setting. Our preliminary results show that only 30% of children achieved histological remission following first-line treatment. The choice of initial therapy varied, although the majority of patients (62.2%) were prescribed a PPI and/or a topical steroid. Our findings highlight the importance of identifying different disease phenotypes to develop personalized therapeutic approaches for the management of EoE.

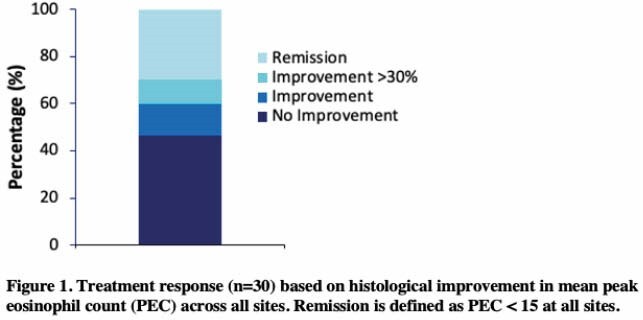

**Funding Agencies:**

SickKids start-up funds (JM Hulst) and the Comprehensive Research Experience for Medical Students (CREMS) Program

**Table:** Demographic and clinical characterization (n=37)

Age, year (median, IQR)	11 (4.5, 12.5)
Sex (% Male)	86.5%
Ethnicity (% Caucasian)	40.9%
Personal history of atopy	91.9%
Family history of atopy	73.0%

